# Resolution of Wing-Beating Tremor and Magnetic Resonance Imaging Lesions in Wilson’s Disease Following Penicillamine

**DOI:** 10.5334/tohm.857

**Published:** 2024-03-04

**Authors:** Igor Vilela Brum, Jacy Bezerra Parmera, Rafael Tomio Vicentini Otani, Izaely Ramos Prates, Leandro Tavares Lucato, Rubens Gisbert Cury, Egberto Reis Barbosa

**Affiliations:** 1 Department of Neurology, Hospital das Clínicas, Faculdade de Medicina da Universidade de São Paulo (HC-FMUSP), São Paulo, SP, Brazil; 2 Institute of Radiology, Hospital das Clínicas, Faculdade de Medicina da Universidade de São Paulo (HC-FMUSP), São Paulo, SP, Brazil; 3 Hospital Israelita Albert Einstein, São Paulo, SP, Brazil

**Keywords:** Tremor, Hepatolenticular Degeneration, Penicillamine

## Abstract

**Background:**

The wing-beating tremor, characteristic of Wilson’s disease (WD), is a disabling symptom that can be resistant to anti-copper and anti-tremor medications.

**Phenomenology Shown:**

This video illustrates severe bilateral wing-beating tremor, moderate head and lower limb tremors, mild cervical dystonia, and subtle cerebellar ataxia, with nearly resolution after penicillamine treatment.

**Educational Value:**

This case highlights a typical aspect of WD, emphasizing the importance of early detection and treatment, and its correlation with MRI findings.

**Highlights:**

This case highlights the typical wing-beating tremor in Wilson’s disease and its correlation with the involvement of the dentato-rubro-thalamic pathway. The early diagnosis and initiation of treatment with penicillamine resulted in an excellent clinical and radiological response.

Neurological symptoms in Wilson’s disease (WD) encompass a broad spectrum, with tremors being a notable manifestation. The characteristic wing-beating tremor in this condition consists of slow, high-amplitude movements in the upper limbs when the patient sustains abduction with flexed elbows and palms facing downward [1]. Herein, we present a remarkable improvement in wing-beating tremor and MRI lesions in a WD patient after penicillamine treatment.

A 27-year-old male presented with a two-year history of worsening upper extremity tremors. Tremors later extended to the lower limbs and head. His symptoms intensified during voluntary movements such as eating, writing, and grooming. Additionally, the patient noted that stress exacerbated the tremors, and there was no response to a trial of propranolol.

Neurological examination revealed a severe wing-beating tremor, mild cervical dystonia, slight dysarthria, and subtle cerebellar ataxia (Video segment 1). Further assessment showed low ceruloplasmin (8 mg/dL; NR: 15–30), elevated 24-hour urinary copper excretion (1187 mcg; NR: <60), and Kayser-Fleischer rings on slit-lamp examination. The diagnosis of WD was established based on the Leipzig score (without genetic testing).

**Video Segment 1:** **Wilson’s disease patient before treatment with penicillamine.** The patient exhibited a severe bilateral wing-beating tremor, moderate head and lower limb tremors, and mild cervical dystonia.

Brain MRI demonstrated symmetric T2/FLAIR hyperintensity in multiple regions (including the dentato-rubro-thalamic pathway), atrophy, and increased iron deposition in the lentiform and caudate nuclei (Figure).

**Figure:**
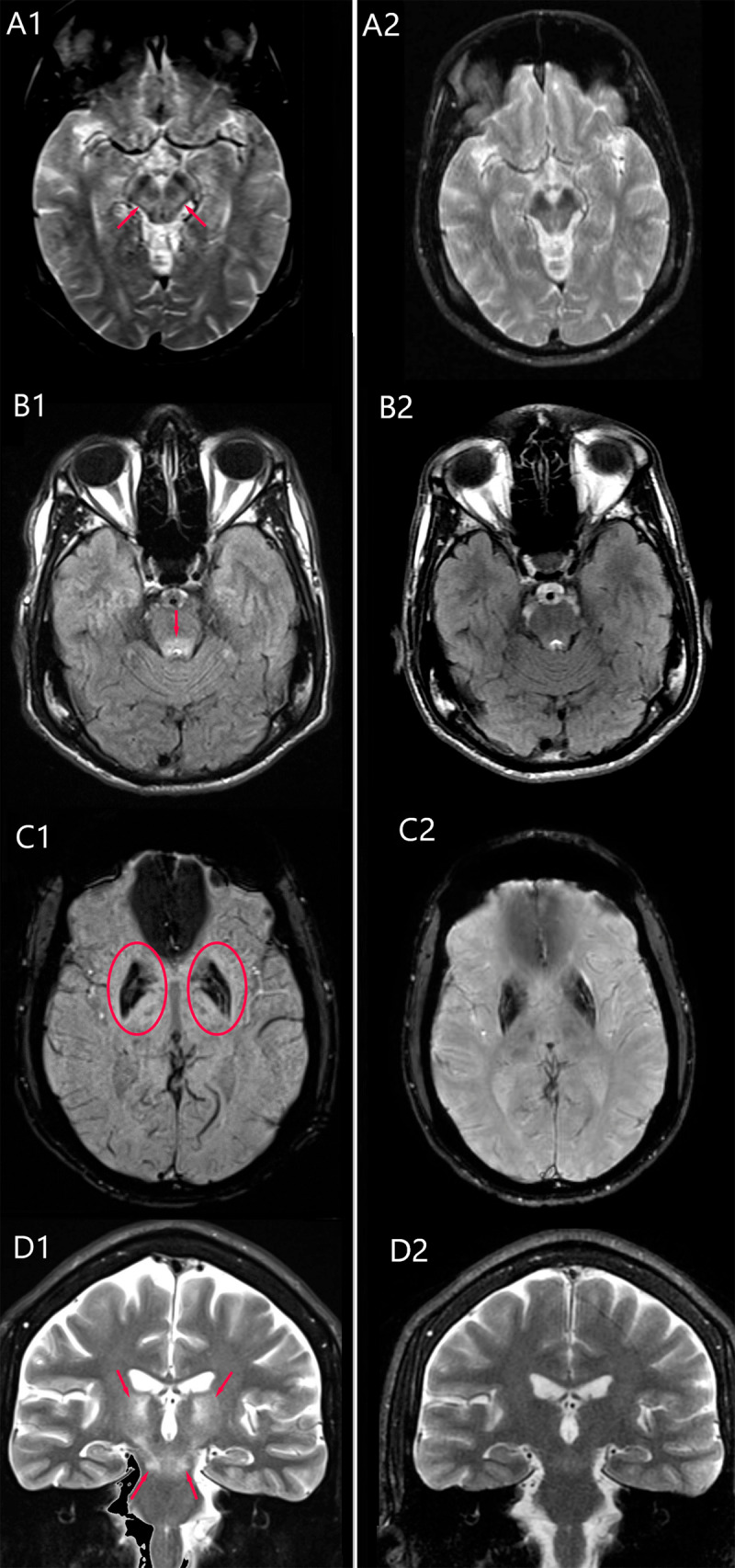
**Left: Wilson’s disease brain MRI before treatment.** MRI shows T2-hyperintensity in the midbrain tegmentum (red arrows in A1), pontine tectum (red arrow in B1), and dentato-rubro-thalamic tract (red arrows in D1). SWI reveals hypointensity and atrophy in the lentiform and caudate nuclei (red circles in C1). **Right: Brain MRI post 3-year penicillamine (A2–D2).** Marked improvement in T2 hyperintense lesions.

Penicillamine was initiated at the dose of 250 mg/day and gradually titrated to 1500 mg/day over a period of six weeks, which led to a remarkable clinical and radiological improvement over the following months. The patient did not receive any symptomatic treatment for tremors. At one-year follow-up, mild tremor and ataxia persisted in the upper limbs, but the wing-beating tremor was no longer observed. The patient gradually regained independence in daily activities and eventually achieved near-complete symptom resolution (Video segment 2). There was marked improvement in MRI lesions with hyperintensity on T2-weighted sequences, while atrophy and hypointensities on SWI remained unchanged (Figure).

**Video Segment 2:** **Wilson’s disease patient follow-up after treatment with penicillamine.** Substantial improvement in symptoms was observed over the three-year follow-up period after penicillamine treatment.

This case emphasizes the characteristic wing-beating tremor in WD and its association with the involvement of the dentato-rubro-thalamic pathway. It also demonstrates the effectiveness of penicillamine in treating this symptom, even though it is traditionally considered resistant to anti-copper and anti-tremor drugs [2,3]. As seen in previous studies, tremor can be a favorable prognostic sign for neurological WD, while its emergence in previously well-managed individuals may indicate suboptimal medication adherence or the need for treatment adjustments [4]. The relatively short time from the onset of symptoms to the initiation of penicillamine treatment may have played a role in achieving an excellent clinical and radiological response in this case.
